# Research on the Mechanisms of Phytohormone Signaling in Regulating Root Development

**DOI:** 10.3390/plants13213051

**Published:** 2024-10-31

**Authors:** Yuru Ma, Ying Zhang, Jiahui Xu, Jiahong Qi, Xigang Liu, Lin Guo, Hao Zhang

**Affiliations:** 1 Ministry of Education Key Laboratory of Molecular and Cellular Biology, Hebei Research Center of the Basic Discipline of Cell Biology, Hebei Collaboration Innovation Center for Cell Signaling and Environmental Adaptation, Hebei Key Laboratory of Molecular and Cellular Biology, College of Life Sciences, Hebei Normal University, Shijiazhuang 050024, China; mayuru916@gmail.com (Y.M.); xjhh0627@163.com (J.X.); guol2024@126.com (J.Q.); xgliu@hebtu.edu.cn (X.L.); linguomail@163.com (L.G.); 2 Institute of Biotechnology and Food Science, Hebei Academy of Agricultural and Forestry Sciences, Shijiazhuang 050051, China; zhh301289@163.com

**Keywords:** phytohormones, auxin, cytokinin, brassinosteroids, root, abscisic acid, peptide hormones

## Abstract

Phytohormones are organic compounds produced in trace amounts within plants that regulate their physiological processes. Their physiological effects are highly complex and diverse. They influence processes ranging from cell division, elongation, and differentiation to plant germination and rooting. Therefore, phytohormones play a crucial regulatory role in plant growth and development. Recently, various studies have highlighted the role of PHs, such as auxin, cytokinin (CK), and abscisic acid (ABA), and newer classes of PHs, such as brassinosteroid (BR) and peptide hormone, in the plant responses toward environmental stresses. These hormones not only have distinct roles at different stages of plant growth but also interact to promote or inhibit each other, thus effectively regulating plant development. Roots are the primary organs for water and mineral absorption in plants. During seed germination, the radicle breaks through the seed coat and grows downward to form the primary root. This occurs because the root needs to quickly penetrate the soil to absorb water and nutrients, providing essential support for the plant’s subsequent growth. Root development is a highly complex and precisely regulated process influenced by various signals. Changes in root architecture can affect the plant’s ability to absorb nutrients and water, which in turn impacts crop yield. Thus, studying the regulation of root development is of great significance. Numerous studies have reported on the role of phytohormones, particularly auxins, in root regulation. This paper reviews recent studies on the regulation of root development by various phytohormones, both individually and in combination, providing a reference for researchers in this field and offering perspectives on future research directions for improving crop yields.

## 1. Introduction

The root systems of plants typically consist of the following three types: primary roots, lateral roots, and adventitious roots. The primary root originates from the embryo and is the first root of the plant; lateral roots arise from root organs, while adventitious roots originate from non-root organs. Different types of roots develop from root founder cells, and the process of their cell division and fate transition is known as root initiation [1,2,3,4,5,6]. During the initiation of different types of roots, auxin is the key phytohormone regulating the initiation of all root types. IAA is primarily synthesized in young leaves and cotyledons and then transported over long distances through the phloem, influencing cellular patterns in the root system [7,8]. IAA is also synthesized in the root apical meristem (RAM) and transported to the upstream adjacent regions of the elongation and differentiation zones through specific proteins (AUX1 and PIN1-7) [9]. Auxin signal transduction is mediated by the transport inhibitor response protein 1 (TIR1)/auxin signaling F-box (AFBs), transcriptional repressors auxin/indole-3-acetic acid (Aux/IAAs), and auxin response factors (ARFs) [3,4,10,11,12]. In the meristem, IAA activates the expression of PLT1-4 transcription factors responsible for maintaining cell proliferation [13,14]. The expression of GRAS family transcription factors SHR/SCR and PLT proteins is necessary for maintaining the SCN and quiescent center (QC), leading to the formation of differentiated root organs [15,16]. The transcription factor lateral organ boundary domain 16 (LBD16), downstream of auxin response factors ARF7 and ARF19, plays a key role in promoting lateral root development [17,18,19,20]. Through LBD transcription factors, cell cycle-related genes like E2Fa and cell wall remodeling genes like EXPANSIN14 are further activated, regulating the initiation and emergence of auxin-induced lateral root development [21,22]. The IAA28-ARF7/ARF19 auxin signaling module regulates the initiation of lateral root founder cells by modulating the GATA23 transcription factor [23]. After auxin establishes the characteristics of founder cells, it triggers lateral root initiation through the SLR/IAA14-ARF7/ARF19 and BODENLOS(BDL)/IAA12-MONOPTEROS(MP)/ARF5 signaling modules. The plasticity of root development is crucial for plants to adapt to their changing environment, and the division ability and timing of root stem cells are critical for precisely establishing root morphology [24,25]. The formation of root tissues originates from the asymmetric division of stem cells surrounding the quiescent center. These stem cells, including columella cell initials, lateral root cap/epidermal cell initials, and cortex/endodermal cell initials, along with quiescent center cells and columella cells, form the SCN. Controlling cell division in the RAM is crucial for maintaining meristem structure stability and proper root growth. In the stele of the meristem, the cell number is determined by the frequency of periclinal cell division, which is controlled by various phytohormones and transcription factors. Auxin and cytokinin positively regulate periclinal vascular cell division in the RAM. The location of the transition boundary from cell division to cell differentiation plays a crucial role in maintaining meristem size and root elongation. In cells surrounding the transition zone (TZ), cytokinin-responsive transcription factor (*ARR1*) can directly activate short hypocotyl 2 (*SHY2*) to inhibit auxin signaling and thus suppress the expression of PIN proteins [26].

CK is a plant hormone originally isolated from maize and other plants or produced synthetically [27,28]. It is primarily synthesized in the roots, in which it promotes CK, thereby supporting tissue differentiation and growth. Working synergistically with auxin, CK regulates cell growth and development in plants. CK, including kinetin (KT), zeatin (ZT), and 6-benzylaminopurine (6-BA), play a key role in cell division and are involved in various physiological processes related to cell growth and differentiation [28,29,30]. Research has shown that in the primary root of Arabidopsis, CK does not directly affect cell division within the meristematic zone but instead influences the size of this zone by modulating the rate of cell differentiation. The application of exogenous CK can reduce the meristematic zone’s size without altering cell division. In mutants deficient in specific genes involved in CK synthesis or signaling, the meristematic zone appears enlarged. Expression pattern analyses and tissue-specific CK depletion experiments suggest that CK specifically acts on vascular tissues in the basal meristematic zone, controlling cell differentiation rates in surrounding tissues through a signaling pathway involving AHK3/*ARR1* and AHK3/*ARR12*. In the primary root’s proximal zone (PZ) of Arabidopsis, the balance between cell division and differentiation is chiefly regulated by the *SHY2* gene, which integrates and modulates the auxin and CK signaling pathways [26,27,28,30,31,32].

BRs are a class of plant-specific steroidal hormones that are essential for plant growth and development [33,34]. Research shows that BR biosynthesis genes are typically expressed at higher levels in young and developing tissues. BR signaling begins when the BR ligand directly binds to the extracellular domain of the plasma membrane (PM)-localized receptor kinase brassinosteroid insensitive-1 (BRI1), triggering a cascade of signaling events. This ultimately alters the phosphorylation status of key downstream transcription factors brassinazole resistant 1 (BZR1) and BR insensitive EMS suppressor 1 (BES1)/BZR2, thereby precisely regulating gene expression and plant growth. Mutants with defects in BR signaling display an increase in the number of vascular cells, indicating that BR acts as a negative regulator of vascular cell division [35,36,37].

Abscisic acid (ABA), also referred to as dormin, is a plant hormone characterized by a sesquiterpene structure [38]. It was named for its role in promoting leaf abscission and is one of the five major natural plant growth regulators [39]. ABA is widely distributed in higher plants and has also been identified in certain plant pathogenic fungi and bacteria [40,41,42]. As a growth-inhibiting hormone, ABA plays a critical role in balancing endogenous hormones and regulating plant growth and metabolism. It is involved in various physiological processes, including flowering, stomatal regulation, stem cell maintenance, fruit ripening, and senescence [43,44,45,46]. ABA, which is known as a stress hormone, plays a central role in plant responses to environmental stress and in regulating the inhibition of lateral root development. Coordination between auxin and ABA signaling pathways is essential for rapid responses to environmental changes, but the mechanisms of their coordinated actions in lateral root formation remain to be explored [47,48].

Plant peptide hormones, which are small molecules consisting of 2–100 amino acids, play crucial roles in regulating cell differentiation, growth, development, and defense mechanisms [49,50]. These peptides function as signaling molecules by binding to extracellular receptors, which, through conformational changes, activate downstream signaling pathways. This process is essential for long-distance intercellular communication and signal transduction, thereby influencing various biological processes, including root development. Based on the presence of an N-terminal signal peptide, peptide hormones are broadly categorized into secretory and non-secretory peptides. Secretory peptides typically contain an N-terminal signal peptide that directs them into the secretory pathway. Structurally, secretory peptides are further classified into the following two types: post-translationally modified peptides, which are derived from the cleavage of larger peptide precursors, and mature peptides, which generally contain 5–20 amino acids. The precursor proteins undergo cleavage by peptidases at the N-terminal signal peptide and subsequent post-translational modifications to form mature peptides. These mature peptides are then packaged into vesicles and secreted from the cell, where they function as signaling molecules [51,52].

This review summarizes the research on the regulation of root development by several plant hormones, both individually and in crosstalk. It aims to lay a foundation for developing a model to elucidate potential interactions between different hormones in the dynamic regulation of normal plant maintenance and offers perspectives on future studies of root development and crop yield improvement.

## 2. Research on Auxin Regulation of Root Development

**Figure 1:**
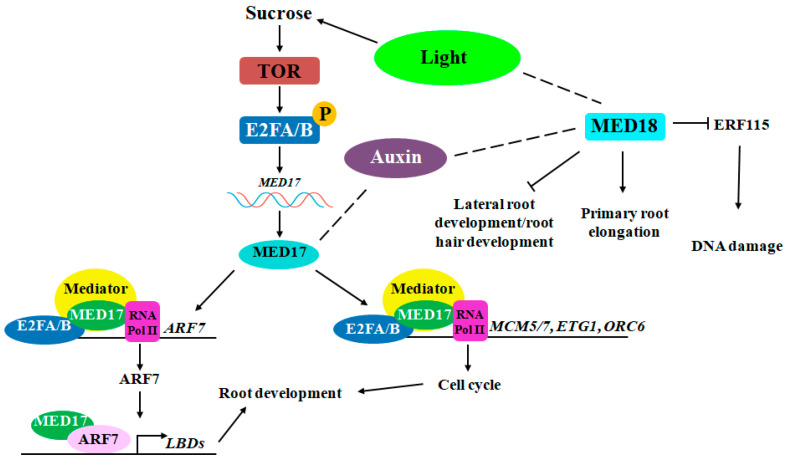
Regulatory network of the auxin involved in regulating root development. Light, energy, and auxin coordinate the regulation of root development.

In *Arabidopsis thaliana*, A-type auxin response factor (A-ARF) genes are closely related to the formation of root organs. The Arabidopsis genome contains five A-ARF encoding genes, which can be divided into the following three subclasses: ARF6 and ARF8 (ARF6/8), ARF7/19, and ARF5 (also known as MONOTEROS, MP). Although these A-ARF genes do not exhibit high expression specificity, their developmental functions are specific [53,54]. Among them, ARF5 controls the initiation of the primary root during embryonic development [17,55], while ARF7/19 controls lateral root initiation [17]. Recent studies have found that ARF6/8 is involved in the initiation of adventitious roots [56]. WOX11 can form a protein complex with ARF6/8, while WOX9 can form a complex with ARF5, indicating that the auxin signaling and the stem cell regulatory pathways need to work closely together to initiate root primordia. This also suggests that the WOX-ARF combinations initiate different types of roots, as follows: WOX11-ARF6/8 are involved in the initiation of adventitious root primordia from founder cells, ARF7/19 are involved in the initiation of lateral root primordia from founder cells, and WOX9-ARF5 are involved in the initiation of primary roots from founder cells [57]. Additionally, sucrose also determines root system architecture, primarily by regulating root meristem activation through the TOR-E2F1-E2FA pathway [58]. Evidence suggests that sucrose can interact with the auxin signaling pathway to jointly regulate root architecture. In Arabidopsis, the mediator (MED) complex is a multi-subunit complex that acts as a bridge between promoter-bound transcription factors and the RNA polymerase II transcription initiation complex, controlling gene expression. Plant MED subunits participate in various developmental processes, hormone signaling pathways, and tolerance to both biotic and abiotic stresses. MED17, through its interaction with sucrose-induced E2FA/B, specifically recruits the MED complex to the promoter of the target gene ARF7, thereby promoting auxin signal transduction and lateral root development. Moreover, MED17 activates cell division in the root meristem by regulating the transcription of cell cycle genes. By integrating sucrose and auxin signaling, MED17 coordinates the development of primary and lateral roots, demonstrating its significant role as a transcriptional processor in root system architecture [59] (Figure 1). MED18 is a key factor for cell viability in the root meristem [60,61]. Loss-of-function mutants (*med18*) exhibit reduced primary root growth but increased development of lateral roots and root hairs [60,61]. This phenotypic change is closely related to enhanced auxin response and transport. Notably, *med18* seedlings exhibit cell death in the root meristem, which intensifies over time or under the influence of DNA damage factors, and the cell regeneration factor ERF115 is highly expressed [60]. Meanwhile, root tip cell death is reduced in *med18* seedlings grown in the dark, but root tip cell death remains unchanged when only the hypocotyl grows under light, indicating that MED18 may protect root meristem cells from localized cell death or respond to root signaling from the hypocotyl under light stimulation [60,61] (Figure 1).

**Figure 2:**
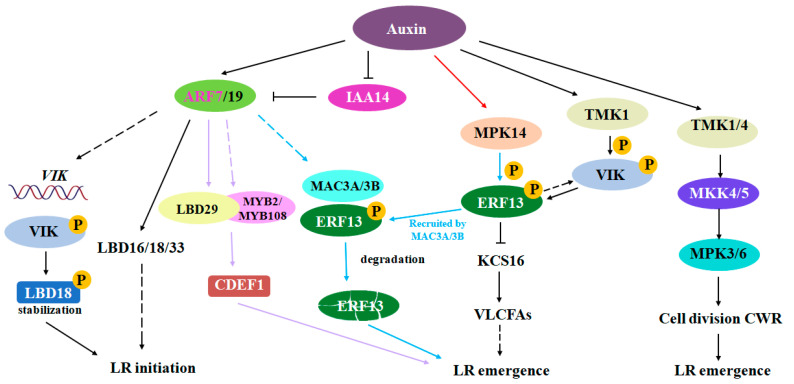
Regulatory network of the auxin involved in regulating lateral root development.

Auxin plays a crucial role in regulating lateral root development. A key component of this signaling pathway is the auxin-responsive factors (ARFs)–lateral organ boundaries–domain transcription factors (LBDs) complex, which regulates almost all stages of lateral root development [17]. It is believed that LBD proteins regulate the transcription of downstream genes in the form of homodimers or heterodimers. Recent studies have identified two MYB transcription factors, MYB2 and MYB108, that interact with LBD29. Both of these factors are induced by auxin in an ARF7-dependent manner. Additionally, the MYB2-LBD29 and MYB108-LBD29 complexes promote the expression of *cuticle destructing factor 1*, a member of the Gly-Asp-Ser-Leu (GDSL) lipase/esterase family involved in LR development [62]. Furthermore, auxin is thought to regulate lateral root development by activating the TMK-MKK4/5-MPK3/6 signaling pathway, which controls the pattern of cell division [63,64]. Research has shown that TMK1 can phosphorylate the VIK protein kinase in response to auxin, and VIK, in turn, regulates lateral root development by phosphorylating ERF13 and LBD18 and cooperates with MPK14 to promote the degradation of ERF13, thereby facilitating lateral root formation. Moreover, VIK and ERF13 form a positive feedback loop that plays a critical role in controlling lateral root development [65]. Studies have found that auxin (1-naphthaleneacetic acid, NAA) can activate not only MPK14 but also MPK1 and MPK2, even in the presence of the auxin receptor transport inhibitor response 1 (TIR1) inhibitor PEO-IAA [66,67]. New research suggests that auxin promotes the synthesis of very long-chain fatty acids (VLCFAs) by activating MPK14 kinase activity, which then phosphorylates ERF13. Phosphorylated ERF13 is degraded by the 26S proteasome, allowing for the expression of downstream KCS16 genes, which promotes the synthesis of VLCFAs. VLCFAs further influence the degradation of pectin in cortical cell walls at the lateral root primordium, affecting the transition from stage IV to stage V of lateral root primordium development, thus impacting lateral root development [68]. Further studies have investigated how ERF13 is degraded. By examining ERF13 interacting proteins (IP-MS), researchers successfully identified the ubiquitin ligases MAC3A/3B as ERF13 interaction partners [69]. A detailed molecular mechanism analysis revealed that although MAC3A/3B can ubiquitinate and degrade ERF13, it requires the following prerequisite condition: MPK14-mediated phosphorylation of ERF13 in response to auxin signaling. Further research confirmed that MPK14-mediated phosphorylation of ERF13 is a crucial signal for enhancing the recruitment of MAC3A/3B and leading to its degradation. Moreover, the study found that auxin not only promotes the transcription of MAC3A/3B but also significantly enhances the stability of MAC3A/3B proteins, thereby synergistically promoting the accumulation of MAC3A/3B proteins during lateral root development and the rapid clearance of ERF13 [15] (Figure 2).

In addition, reactive oxygen species (ROS) and auxin regulate root development by influencing cell division and differentiation. A study identified a mutant, *dpr2*, with defects in maintaining RAM. The *DPR2* gene encodes a phosphoethanolamine N-methyltransferase, PEAMT1, which catalyzes the biosynthesis of phosphocholine in Arabidopsis [70]. The loss-of-function of the PEAMT1 gene affects the root SCN, meristem zone, elongation zone, and differentiation zone, leading to the consumption of the RAM. Additionally, the *dpr2* mutant shows defects in the amount, polar distribution, and endocytosis of PIN proteins in the root tip. The accumulation of excess hydrogen peroxide and auxin in the elongation and differentiation zones of the primary root in the *dpr2* mutant accelerates cell differentiation, leading to the consumption of the RAM. After phosphocholine starvation treatment, inhibiting the excessive accumulation of ROS or auxin signaling in the *dpr2* mutant can partially prevent the differentiation of the RAM [70].

Melatonin is a novel plant signal that regulates root architecture in many plant species, including primary root elongation, adventitious root formation, and lateral root development [71,72,73,74]. An increasing number of studies have shown that melatonin is an important factor in improving plant resistance to abiotic stress and that the effect of melatonin at hormonal levels on root growth is related to the regulation of auxin signaling [75]. Recent studies have revealed that exogenous melatonin significantly alleviates the inhibitory effect of hypoxia stress on rice root growth. A morphological–phenotypic analysis showed that after melatonin pretreatment, under hypoxia stress, the length of primary roots, lateral roots, and lateral root density increased by 11.6%, 8.2%, and 36.8%, respectively. This indicates that melatonin pretreatment significantly increased the activity of superoxide dismutase (SOD) in seedling roots, reduced the accumulation of superoxide anions (O^2^•−), and increased the content of hydrogen peroxide (H_2_O_2_) under hypoxic stress. This study demonstrated the important role of melatonin in regulating root growth under hypoxic stress, providing a new strategy to enhance hypoxia tolerance [76].

## 3. Cytokinin Regulation of Root Development

**Figure 3:**
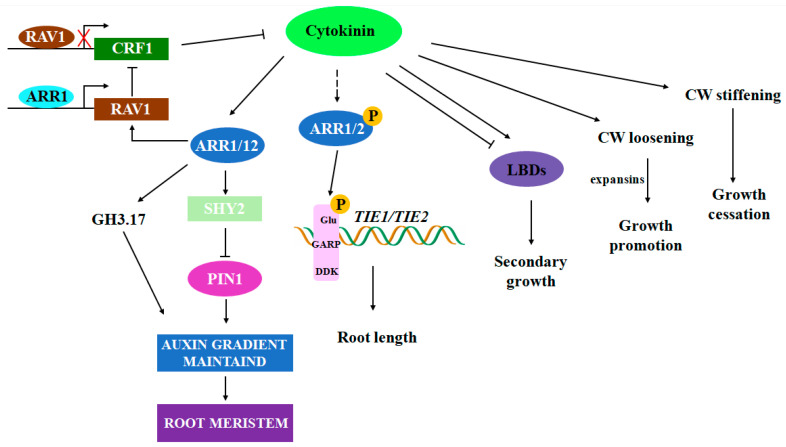
Regulatory network of the CK involved in regulating root function and maintenance.

Previous research has shown that cytokinin signaling is one of the key hormones controlling root development. The absence of B-type ARR, a crucial regulatory factor in cytokinin signaling, leads to a long-root phenotype. A recent study identified a mutant, *rav1*, that also exhibits significantly longer primary roots with a markedly expanded meristem containing more cells [12]. At the transcriptional level, cytokinin response genes such as *ARR1* and *ARR12* are significantly downregulated in the mutant roots, indicating a defect in cytokinin signaling. Moreover, cytokinin-induced Aux/IAA gene *SHY2* and the auxin efflux gene PIN1 are regulated in the *rav1* mutant, resulting in altered auxin transport and distribution, which affects the size of the meristem. Further research has revealed that cytokinin can upregulate *RAV1* expression during post-embryonic root development via *ARR1*. The regulation of *RAV1* expression is part of a secondary cytokinin response, which ultimately inhibits *CRF1* expression, thereby enhancing cytokinin signaling [77]. In other plant hormone signaling pathways, such as auxin and abscisic acid, a common negative feedback regulation involving a transcription factor with an EAR motif is used to precisely regulate hormone signals. Similarly, in the cytokinin signaling pathway, recent research has found that TIE2 serves as a bridge, interacting with B-type ARR at its N-terminus and connecting the EAR motif at its C-terminus with TPL/TPR corepressors. The double mutant *tie1 tie2* exhibits loss-of-function, leading to the release of B-type ARR repression and an excess of cytokinin signaling, which results in shorter roots [78] (Figure 3).

In Arabidopsis roots, the boundary between cell division and differentiation relies on the antagonistic interaction between cytokinin and auxin, particularly in the formation of the TZ. Recent studies have shown that cytokinin can both activate *GH3.17* transcription through *ARR1* to regulate auxin degradation and inhibit auxin transport by *SHY2*, ultimately regulating auxin distribution in the root tip and forming a zone of minimum auxin concentration. This minimal zone acts as a signal, determining the TZ’s location and initiating differentiation in the meristematic cells [79]. With the functional partitioning of the adventitious root meristem, cytokinin and auxin distributions exhibit complementary patterns, as follows: auxin is confined to the root apical stem cell niche, while cytokinin mainly accumulates in the root cap and vascular tissues. Cytological observations show significant defects in vascular tissue and root cap differentiation in the adventitious root meristem of *ahk2/3/4* and *arr1/10/12* mutants, with marker genes of the stem cell niche being misexpressed in the root cap and vascular tissues [80]. This suggests that during the development of adventitious root primordia into adventitious root meristems, cytokinin may antagonize the distribution of auxin, confining auxin to the root apical stem cell niche and promoting differentiation of the root cap and vascular tissues. Overall, cytokinin plays two critical roles in promoting root differentiation, facilitating the transition of the meristem into the rapid growth phase and inhibiting growth. This mechanism requires the action of the auxin efflux carrier AUX1 to increase auxin activity in the elongation zone [81]. Cytokinin regulates vascular development during both primary and secondary growth. During the primary growth of Arabidopsis roots, cytokinin and auxin jointly promote the proliferation of the procambium cells and the formation of vascular tissue. In the absence of cytokinin, secondary growth cannot be initiated. Conversely, increased levels of cytokinin can promote secondary growth. Cytokinin directly activates the transcription of *LBD3* and *LBD4*, whereas *LBD1* and *LBD11* are induced only after prolonged cytokinin treatment. Genetic studies have revealed a two-stage mechanism downstream of cytokinin signaling. *LBD3* and *LBD4* regulate the activation of secondary growth, while *LBD1*, *LBD3*, *LBD4*, and *LBD11* collectively promote the radial growth and maintenance of the cambial stem cells. Overexpression of *LBDs* promotes rapid cell growth and division, accelerating secondary growth. Further analysis found that LBDs can rapidly suppress cytokinin signaling through negative feedback to maintain the balance of root secondary growth [82] (Figure 3). C-terminal coding peptide (CEP) is a key signaling mechanism for plants to adapt to different external environments and intercellular communication, and a variety of environmental factors and nutrients affect the expression of CEP signaling pathway genes. The CEP and trans-zeatin (tZ, a plant cytokinin) pathways may interact with CEPD glutaredoxin. On the plant ground, the production of root CEP promotes an increase in CEPD1,2 content. Subsequently, CEPD1,2 is transmitted through the phloem to the roots. In roots, tZ-type cytokinins induce CEPD1 production through AHK2,3 signaling transcription and may induce CEPD2 production through other mechanisms, such as post-translational mechanisms. The CEPD of aboveground and underground parts ultimately combines root growth response with plant nutrition and environmental stress [83].

## 4. BR Regulates Root Development

In recent years, with the combination of genetics, mass spectrometry, and isotope labeling technologies, the BR biosynthetic network has been successfully established, outlining a series of biosynthetic steps and organizing the positions of biosynthetic enzymes in a linear sequence [84]. Previous reports have confirmed that BRs do not undergo long-distance transport [85]. In Arabidopsis, BR signaling controls cell division and cell elongation by establishing a signal gradient along the longitudinal root axis [86]. By comparing the BR content in the meristematic and elongation zones of pea roots, it was found that the elongation zone had significantly higher BR levels than the meristematic zone, which is consistent with the spatial distribution of biosynthetic enzyme expression and signal intensity. This indicates that the coordinated growth of roots requires a gradient distribution of BR concentration, with lower hormone levels in the meristematic tissue and higher levels in the elongation zone, which are attributable to the spatial distribution of biosynthesis. Plants induced with BR-deficient phenotypes, along with weakened BR signal strength, further demonstrate the possibility of short-distance transport of BRs between cell layers [87]. The maintenance of the root meristematic state depends on the coordination of multiple hormone signals, with the interaction between BR and auxin playing a crucial role. BR not only promotes auxin synthesis but also inhibits auxin signal output, playing a complex and essential role in maintaining the root meristem. Recent research has shown that blocking BR-induced auxin synthesis leads to the rapid depletion of the root meristem mediated by BR, revealing that BR maintains meristem function by promoting auxin synthesis. Conversely, reducing BR levels can help plants resist severe losses in auxin biosynthesis, maintaining the morphology of the meristem and further highlighting the critical role of BR in regulating auxin signaling. Interestingly, when both auxin and BR synthesis are inhibited, the meristem can regenerate after root damage, relying on local auxin synthesis. This finding suggests that the balance between BR and auxin is crucial for the regenerative capacity of the root meristem. Using BIN2 to selectively inhibit BR signaling produces different meristem phenotypes depending on the type of tissue disrupted, which is similar to BR-deficient mutants or plants treated with high levels of BR, indicating that the interaction between BR and auxin differentially regulates the root meristem in various tissue layers [88].

**Figure 4:**
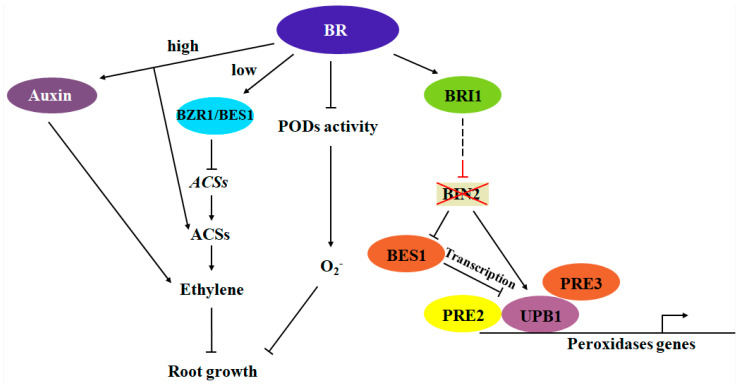
Regulatory network of the BR crosstalk involved in regulating root growth.

The *det2-9* mutant, which is defective in BR synthesis, exhibits a short-root phenotype. The short-root phenotype of the *det2-9* mutant can be partially rescued by *det2-9/acs9* double mutant and *det2-9/ein3/eil1-1* triple mutant, which are defective in ethylene synthesis and signaling, respectively. Unlike BR’s role in stabilizing ACS proteins to induce ethylene biosynthesis, research has found that BR signaling transcription factors BES1 and BZR1 can directly bind to the promoter regions of the ACS7, ACS9, and ACS11 genes to suppress their expression, indicating a natural regulatory mechanism at physiological BR levels. Additionally, compared to the wild type, the *det2-9* mutant exhibits the excessive accumulation of superoxide anions, and increased levels of superoxide anions inhibit root growth. BR controls superoxide anion accumulation mainly through the peroxidase pathway rather than the NADPH oxidase pathway. This study reveals the hormonal cross-regulation among BR, ethylene, and ROS in controlling root growth and development in Arabidopsis [89]. Moreover, in the absence of BR, BIN2 phosphorylates UPB1, promoting UPB1 protein stability and transcriptional activity and enhancing UPB1’s regulation of downstream genes, thereby negatively regulating root meristem development. When BR is perceived, BIN2 activity is inhibited, and BES1 regulates UPB1 expression at the transcriptional level, promoting root meristem development [90]. HD-ZIP III class transcription factors positively regulate BR biosynthesis-related genes in vascular tissues. Genetic and physiological analyses of HD-ZIP III mutants and BR biosynthesis genes indicate that BR biosynthesis regulated by HD-ZIP III is a key factor in inhibiting periclinal cell division around vascular cells. BR induces the division of vascular cells in RAM by inhibiting cytokinin responses [91] (Figure 4).

Faced with the complex living environment in the soil, plant roots must not only absorb nutrients but also block harmful stresses. The Casparian strip in the endodermis and the xylem in the vascular bundle are crucial for the selective absorption and transport of water and nutrients. The formation of these two important structures requires lignin synthesis and accumulation. However, root lignification in plants only occurs when the root cells mature. Studies have found that BR signaling has an antagonistic relationship with SHR in the initiation of root lignification [92]. Further research has shown that the key transcription factor regulating BR signaling, BZR1, hinders the activation of downstream lignification-related genes by binding to the SHR protein. Short-root (SHR), a key factor in endodermal formation, has been found to not only regulate the formation of the Casparian strip in the endodermis but also promote the expression of genes involved in xylem formation. However, SHR expression remains uniform throughout root development. To prevent premature lignification of root cells before maturity, it is likely that there are antagonistic factors that collaborate with SHR to regulate the lignification process in roots [92]. Interestingly, BR signaling itself also fluctuates during root development. As root cells enter the rapid elongation zone, BR signaling intensifies, and BZR1 expression peaks. Then, as root cells enter the maturation zone, BZR1 expression drops sharply, coinciding with the spatiotemporal initiation of root lignification [92]. Additionally, the study found that plant hormones such as ABA, auxin, and ethylene play roles in antagonizing BR signaling during root development, collectively coordinating the lignification process in plant roots [92]. BR receptor mutants *bri1-119* and *bri1-301* exhibit lower sensitivity when faced with reduced boron supply, while gain-of-function mutants bes1-D and *pBZR1-bzr1-D* show insensitivity under low boron stress conditions. Boron deficiency hinders BR accumulation, thereby downregulating BR signaling and modulating the root elongation process. This may be due to the reduced expression levels of *BR6ox1* and *BR6ox2* mRNA caused by boron deficiency [93] (Figure 4).

## 5. ABA’s Role in Root Development

The *hab1-1abi1-2abi2-2pp2ca-1* quadruple mutant (*Qabi2-2*), which lacks the key negative regulators in the ABA signaling pathway—clade A protein phosphatases type 2C (PP2Cs)—exhibits a significant increase in proton concentration in the apoplast of its roots, leading to enhanced root growth under both normal and water-stress conditions. Low concentrations of ABA (0.1 micromolar) can promote root development by inhibiting PP2C, thereby increasing hydrogen ion levels in the apoplast of the roots, a phenotype very similar to that of the *Qabi2-2* mutant [94]. Additionally, the *Qabi2-2* mutant exhibits hydrotropic root growth characteristics, which are related to the proton gradient in the apoplast of its root elongation zone. In Arabidopsis, ABA-insensitive 1 (a key PP2C in ABA signaling) can interact with the plasma membrane-localized AHA2, leading to the dephosphorylation of Thr947 and subsequently inhibiting AHA2 activity. ABA promotes root development by inhibiting this process [94].

The transcription factor ERF1 is a key integrator of environmental signals in Arabidopsis. ERF1 promotes local auxin accumulation and alters its distribution by upregulating PIN1 and AUX1 while suppressing ARF7 expression, thereby inhibiting lateral root formation [1]. Meanwhile, ABA-responsive ERF1 mediates the crosstalk between ABA and auxin signaling pathways to regulate lateral root initiation in Arabidopsis. ABI3 is a negative regulator of lateral root formation that activates ERF1 expression by binding to its promoter. Conversely, ERF1 can also activate ABI3, forming a regulatory loop that can rapidly amplify the signal. Further research has shown that the interaction between ABI3 and ERF1 reduces their binding activity to cis-regulatory elements, thereby weakening the expression of genes involved in lateral root formation and ABA signaling pathways, such as PIN1, AUX1, ARF7, and ABI5. This interaction may provide a molecular rheostat in the plant’s internal environment to prevent the excessive amplification of auxin and ABA signals [95].

K^+^ efflux antiporters (KEAs) belong to the cation proton antiporter family 2 and are present in various organisms. In Arabidopsis, KEA1 and KEA2 have a high sequence similarity (84.5%) and are localized to the inner membrane of chloroplasts, in which they mediate osmoregulation and pH balance, playing a vital role in plastid development and seedling growth. However, the functions of KEA1 and KEA2 during the early developmental stages of seedlings are not well understood. Recent studies have shown that KEA1 and KEA2 are essential for primary root growth. When wild-type and kea1kea2 mutant roots were treated with the photosynthesis inhibitor DCMU, which blocks the synthesis of endogenous sucrose, it was observed that sucrose could promote root growth, and KEA1 and KEA2 indirectly influence primary root growth by regulating photosynthesis. The study also found that the absence of KEA1 and KEA2 reduces the levels of the hormones ABA and IAA, indicating that KEA1 and KEA2 are involved in primary root growth in an ABA-dependent manner. ABA stimulates root growth at low concentrations but inhibits it at high concentrations [78].

## 6. Peptide Hormones Regulate Root Development

Peptide hormones are peptides with hormone activity. Both auxin and plant peptide hormones regulate multiple aspects of plant growth and development, and there is crosstalk between auxin and plant peptide hormones [96]. Root meristem growth factors (RGFs) are a class of peptide hormones unique to plants, playing a crucial role in the development of the root apical meristem. Arabidopsis has 11 RGF members, with RGF1/2/3 being expressed at the root tip and involved in maintaining SCN [97]. RGFs are recognized by five functionally redundant receptor-like protein kinases—RGF1 insensitve 1 (RGI1) to RGI5—which induce the expression of downstream apetala 2 (AP2) transcription factor genes—plethora 1 (PLT1) and PLT2, and participate in the regulation of PLT2 protein stability [96,98,99]. Peptide hormones regulate root development. The RGF1 peptide is crucial in controlling the size of the meristematic zone, functioning as both an intrinsic and extrinsic signal. Treating roots with RGF1 increases the size of the meristematic zone, while the *rgf1/2/3* triple mutant of *Arabidopsis thaliana* shows a reduced meristematic zone. The downstream transcription factor RITF1 is induced by RGF1 in the meristematic zone. RITF1 can regulate ROS signaling and the size of the root meristem downstream of the RGF1 pathway. ROS regulates the stability of PLT2 protein by modulating O_2_^−^ and H_2_O_2_ levels [100]. Five closely related leucine-rich repeat receptor-like kinases (LRRRLKs) named Rgf1 receptors (Rgf1 insensitives, RGIs) or Rgf1 receptors (RGFRs) sense Rgf1 signals and redundantly control the maintenance of the root stem cell niche. The quintuple mutant of *rgf1* receptors (*rgfr*) lacks most cells in the root meristem and is insensitive to *rgf1*. BRI1-associated receptor kinase 1 (BAK1) and its paralogous genes somatic embryogenesis receptor-like kinases (SERKs) can function as coreceptors for RGIs, but experimental evidence is still lacking. Recent studies have indicated that the root length of single, double, and triple mutants of SERKs is shorter, and their response to RGF1 is significantly reduced. RGIs can interact with BAK1 and become phosphorylated, which is a process that is dependent on RGF1. Moreover, RGF1 can activate MAPK through RGIs and SERKs. This confirms that RGIs play a crucial role in regulating root apical meristem development [101]. Specifically, through which factors does RGF1-RGI1 regulate the stability of PLT proteins? A recent study found that after treating Arabidopsis seedlings with Rgf1, mitogen-activated protein kinase 4 (MKK4) and MAP kinase 3 (MPK3) co-precipitate with RGI1-FLAG. Genetic and biochemical analyses confirmed that MKK4 and MKK5, along with their downstream targets MPK3 and MPK6, are essential RGI-dependent regulatory factors for root meristem development. Additionally, the MKK4/MKK5-MPK3/MPK6 module functions downstream of the MAPKKK gene YDA. This study indicates that Rgf1-RGI1 regulates the expression of PLT1/PLT2 through the YDA-MKK4/MKK5-MPK3/MPK6 signaling cascade [102]. In addition to RGF1 and its receptors, new members of the peptide hormone family have recently been identified. A new member of the plant peptide hormone family, PIP2, is induced by auxin, whereas PIP1 and PIP3 are not. The overexpression of PIP2 in plant seedlings results in slightly shorter root lengths and longer hypocotyls compared to wild-type Col-0. This study reveals its role in regulating the elongation of roots and hypocotyls in *Arabidopsis thaliana* [103].

## 7. Conclusions and Perspective

The study of plant root tip cells has long been a significant area of research for plant biologists. By investigating the differentiation and development processes of plant root tip cells, a better understanding of plant growth and development mechanisms can be achieved, providing more detailed and accurate data for plant breeding and plant biotechnology research [104]. Auxin plays a crucial regulatory role in plant growth and development. Auxin can also influence root system development, increasing root branching and growth, thereby enhancing the plant’s ability to absorb nutrients and water [105,106]. Gibberellins (GA) are a class of diterpenoid plant hormones that play essential roles throughout the plant’s life cycle, promoting cell division and elongation, seed germination, hypocotyl and stem elongation, root growth, and flowering [107,108,109,110]. Studies have shown that exogenous low-concentration GA treatment can increase the total root length of soybean, but as the concentration increases, the growth of the primary root, lateral roots, and total root length is inhibited, and the root diameter is significantly reduced. Drought stress affects the expression of GA metabolism-related genes in rice roots [111]. Gibberellins typically interact with other hormones to regulate root growth. Studies have shown that ethylene and gibberellins are involved in regulating primary root development in rice seedlings. As the primary root of rice seedlings grows, it promotes ethylene synthesis and the accumulation of OsEIL1 protein. The accumulated OsEIL1 protein further activates ABA synthesis genes *MHZ4/MHZ5*, auxin synthesis gene *OsYUC8*, and gibberellin metabolism genes *OsGA2ox1*, *2*, *3*, and *5*, leading to elevated ABA and auxin levels in the roots while reducing the content of active gibberellins, ultimately causing the primary root to stop growing [112]. The primary regulatory mechanisms of plant hormones in root development have been elucidated, but the regulatory networks involving interactions among various plant hormones require further refinement [113]. Some regulatory mechanisms remain unclear. In-depth research on hormone-regulated root development mechanisms not only lays a theoretical foundation for refining root development mechanisms but also has significant implications for improving crop yields. As functional genomics research in crops advances, clearer mechanisms of plant root development will be elucidated, greatly enhancing our understanding of root development. The improvement of root breeding capabilities driven by root functional genomics research will continuously break through the bottlenecks of traditional breeding, leading to a revolution in crop breeding. This will be of great significance in cultivating resource-efficient and environmentally friendly agricultural production systems and enhancing international competitiveness in biotechnology-centered breeding [114,115,116].

## Data Availability

The data are available in the article.
